# “Letting Go” (Implicitly): Priming Mindfulness Mitigates the Effects of a Moderate Social Stressor

**DOI:** 10.3389/fpsyg.2016.00872

**Published:** 2016-06-13

**Authors:** Catherine M. Bergeron, Isabelle Almgren-Doré, Stéphane Dandeneau

**Affiliations:** Department of Psychology, Université du Québec à MontréalMontreal, QC, Canada

**Keywords:** mindfulness, implicit processes, stress reactivity, stress perceptions, cortisol

## Abstract

This experimental study investigated whether implicitly priming mindfulness would facilitate psychological and cortisol recovery after undergoing a standardized psychological stressor. After completing baseline measures of well-being, all participants (*N* = 91) completed a public speaking stress task, were implicitly primed with “mindfulness” or “neutral” concepts using a scrambled sentence task, and finally, reported their situational well-being and provided cortisol samples. Simple moderation regression analyses revealed that the implicit mindfulness condition had significant beneficial effects for participants with low trait mindfulness. These participants reported higher situational self-esteem as well as less negative affect, perceived stress, and self-reported physiological arousal than their counterparts in the control condition. Cortisol analyses revealed that participants in the implicit mindfulness condition, regardless of level of trait mindfulness, showed a greater decline in cortisol during the early recovery stage compared to those in the control condition. Overall, results suggest that implicitly activating mindfulness can mitigate the psychological and physiological effects of a social stressor.

## Introduction

The topic of mindfulness, its benefits, and the multiple ways it is practiced, has gained popularity over the last few decades. To this end, defining the concept and specifying its core components has been an important step. A commonly agreed upon definition of mindfulness is that it refers to a state of mind where one is “paying attention in a particular way; on purpose, in the present moment, and non-judgmentally” (Kabat-Zinn, 1994, p.4). Although commonly viewed as an exercise of working toward a “mindful state of mind” through repeated practice (e.g., mindfulness-based meditation, mindfulness breathing), mindfulness is also, according to some authors, inherently a state that can vary both within persons and between individuals (Brown and Ryan, 2003). In this sense, mindfulness also refers to individual differences in people’s inherent predisposition to mindfulness (i.e., *trait* mindfulness). Finally, it has also been suggested by some authors that mindfulness is a capacity present to some degree in all people, regardless of level of past training or practice (Goldstein, 2002; Brown and Ryan, 2003; Kabat-Zinn, 2003; Brown et al., 2007).

In recent years, research on the mitigating effects of trait mindfulness on stress has garnered much attention (e.g., Feltman et al., 2009; Gilbert and Christopher, 2010; Graham et al., 2013; Creswell and Lindsay, 2014; Creswell et al., 2014). Arch and Craske (2010) for example showed that trait mindfulness moderates stress responses of clinically anxious and non-anxious people. Similarly, Brown et al. (2012) showed that higher dispositional (trait) mindfulness is associated with lower cortisol reactivity following the Trier Social Stress Test (TSST), a standardized laboratory stressor (Kirschbaum et al., 1993). Stemming from the idea that all humans possess an innate capacity for mindfulness, we propose that this capacity could be triggered or activated through standard priming techniques. In an experimental study, Williams et al. (2009) investigated whether people’s innate capacity for cognitive reappraisal (e.g., carefully analyze, reassess) could be implicitly activated and showed that participants implicitly primed with cognitive reappraisal experienced a decrease in heart rate during a stressful task to a similar extent as those explicitly instructed to cognitively reappraise the stressful task. In addition, results for the non-conscious activation of cognitive reappraisal were most pronounced for those *low* in trait reappraisal, that is, for those who do not habitually use reappraisal strategies (Williams et al., 2009). Based on Williams et al.’s (2009) findings, we propose that people’s innate capacity for mindfulness may be activated via unconscious and automatic processes (e.g., Bargh and Chartrand, 1999; Dijksterhuis and Nordgren, 2006) and show positive stress-mitigating effects. Therefore, in the current study, we set out to test whether implicitly activating people’s innate capacity for mindfulness with a semantic priming procedure would mitigate the psychological and physiological effects of a moderate social stressor. Whereas others, such as Williams et al. (2009) have investigated how implicitly activating cognitive reappraisal *prior* to a stress influences its perception and experience, we instead we set out to investigate how implicitly activating mindfulness *after* a stress would influence one’s recovery after the stress experience. Thus, we expected that priming mindfulness after experiencing stress would *mitigate* its effects during the recovery stage. In addition, we set out to test whether the effect of implicitly activating mindfulness would be moderated by people’s level of trait mindfulness. More specifically, research has shown higher trait mindfulness is associated with a variety of positive self-regulatory and emotional well-being processes (Brown and Ryan, 2003), suggesting that participants *low* in trait mindfulness would arguably benefit from engaging in such positive self-regulatory processes. Therefore, in the current study, we tested whether participants with low trait mindfulness, namely people who do not engage in mindfulness related regulatory strategies, would most benefit from having their innate capacity for mindfulness implicitly activated. We anticipated that individuals with high levels of trait mindfulness would naturally respond adaptively to a stressful experience, whereas individuals with low trait mindfulness would benefit from a temporary activation of their innate capacity for mindfulness.

## Materials and Methods

### Participants

Based on similar studies (namely Williams et al., 2009), the effect size estimate in the current study was approximated at Cohen’s *f*^2^= 0.09 (in the small to medium range). Thus, with an alpha level of 0.05 and a power of 0.80, G^∗^Power’s linear multiple regression sample size calculator suggests a total sample of 90 participants. Data collection was stopped at 94 participants. Due to technical difficulties, data from three participants were excluded from analyses. Our final sample consisted of 91 undergraduate students (71 females, *M*_age_ = 24.89 years, *SD*_age_ = 6.10) at the Université du Québec in Montréal. Participants were recruited by email with an invitation to participate in a 90-min study on ‘non-verbal behavior’ in exchange for a 25$ compensation. All participants met the inclusion criterion of being proficient in French.

### Materials and Procedures

The study consisted of two sessions: an online pre-test and a laboratory session. This study was approved by UQAM’s ‘Comité d’éthique de la recherche pour les projets étudiants impliquant des êtres humains’. All subjects gave written informed consent in accordance with the Declaration of Helsinki.

During this pre-test session, participants completed the French version of the Kentucky Inventory of Mindfulness Skills (KIMS; Baer et al., 2004; French validation: Nicastro et al., 2010, α = 0.79) as a measure of trait mindfulness. Participants also provided information about behaviors known to influence cortisol secretion such as medication use, whether they smoked and average number of cigarettes they smoked per day. These measures were used as covariates in the cortisol analyses. At the end of the online session, participants received a subject number and were scheduled for their laboratory session an average of 1 week later. This online pre-test session was conducted separately from the laboratory session to shorten the laboratory session, and most importantly, to prevent mindfulness scales (e.g., items from the KIMS) from activating mindfulness concepts *prior* to our implicit activation of mindfulness task during the laboratory session.

Laboratory sessions were run between 1:00 and 4:45 p.m. to control for the pronounced circadian variation in cortisol secretion (Dickerson and Kemeny, 2004). The laboratory session started with an informed consent followed by a question asking participants the time they had their last coffee (used as covariate in the cortisol analyses). Participants then completed baseline measures of well-being that consisted of French versions of the following scales: situational self-esteem, measured with the 4-item Rosenberg Self-esteem scale adapted to ask about *current* feelings of self-esteem (Major et al., 1998; French validation: Vallières and Vallerand, 1990; α = 0.69), situational perceptions of stress, measured with the 4-item Perception of Stress Scale (Cohen et al., 1983; French validation: Bellinghausen et al., 2009; α = 0.72), positive and negative affect using the 10-item Positive and Negative Affect Schedule (Watson et al., 1988; French validation: Gaudreau et al., 2006; α = 0.73 and 0.57, respectively), and finally an *indirect* measure of negative emotions using the self-reported physiological arousal scale (Hess and Blairy, 2001; original scale in French, α = 0.65) which asks participants to report a variety of physiological sensations (e.g., sweaty palms, accelerated heartbeat). Afterward, participants were asked to rest while reading magazines for 5 min. The aim of this rest period was to ensure that participants felt acclimatized to the laboratory context before taking their baseline measure of cortisol.

Following the baseline measures and resting period, participants were then told that the next task consisted of a mock job interview and were given instructions for the public speaking component of the Trier Social Stress Task (Kirschbaum et al., 1993; see Supplementary Material for description of the public speaking task). After the speech task, participants indicated their post-stressor levels of positive and negative affect, and self-reported physiological arousal. They were then assigned to either the mindfulness (even-numbered subjects) or control priming condition (odd-numbered subjects). Participants in the mindfulness condition were primed with mindfulness words via a modified version of the scrambled sentences task (Srull and Wyer, 1979). This task consisted of rearranging words into a meaningful sentence (e.g., ‘is awareness delicious espresso’ becomes ‘espresso is delicious’). The *unchosen* word (or words), that is the word that does not become part of the finished sentence, were used as primes to activate mindfulness (e.g., *awareness* in the previous example). Seeing as the goal of the task is to construct a meaningful sentence with the available words, participants’ attention and focus is almost exclusively drawn to the non-prime words while being unobtrusively presented with prime words (e.g., focusing on ‘espresso is delicious’ while being exposed to ‘awareness’). In the experimental condition, participants unscrambled eight sentences containing mindfulness primes (original *French primes*/English translation were: *moment présent*/present moment*, laisser-aller*/let go, *sans jugement*/non-judgmental, *instant présent*/present instant, *lâcher prise*/letting go, *détachement/*detachment, *présence attentive*/attentive presence, *acceptation*/acceptance) and four sentences containing neutral primes. In the control condition, participants completed 12 sentences with neutral prime words (e.g., table, shoe, and house). The words and procedure used to implicitly prime mindfulness underwent an initial validation process conducted with a separate group of 41 independent evaluators (see Supplementary Material for a full description of the validation procedure). Results of this validation showed that mindfulness words were rated as significantly less positive than positive words and more negative than neutral words, indicating that the set of mindfulness prime words do not prime positivity *per se*. In addition, the set of mindfulness words were rated as significantly more related to mindfulness dimensions (e.g., acceptance, attentiveness, observing) than non-mindful dimensions (e.g., resistance, inattentiveness, denying).

After the priming procedure, participants were instructed to sit quietly for 10 min. The purpose of this rest period was to provide participants with an opportunity to reflect on their speech performance. After the rest period, participants provided a brief description of their thoughts during the 10-min rest period and then completed outcome measures of well-being (situational self-esteem, α = 0.80; perception of stress, α = 0.72; positive and negative affect, α = 0.72 and 0.70, respectively; and self-reported physiological arousal, α = 0.67). To make efficient use of participants’ presence in the laboratory during the 40-min post-speech time period, participants were asked to complete a classic Stroop task as part of a separate line of investigation exploring the cognitive effects of activating mindfulness implicitly (e.g., Bergeron et al., unpublished manuscript). All participants completed the same color-naming Stroop task, which took approximately 5 min, and the data were not part of the main objective of the current analyses. Participants then read magazines until the 40-min post-speech time point. All participants were verbally debriefed at the end of the experiment and thanked for their participation.

Throughout the laboratory session, saliva samples were collected with salivette sampling at five time points: T1 – baseline (after baseline measures of well-being and rest period); T2 – immediately after the speech task; T3 – 10 min post speech and immediately after priming procedure; T4 – 25 min post speech and after outcome measures of well-being; and T5 – 40 min post speech. All salivette samples were kept in a refrigerator at 4°C until they were sent for biochemical analysis.

### Main Analyses and Results

Preliminary analyses indicated that baseline levels of situational well-being (i.e., self-esteem, negative affect, self-reported physiological arousal, and perceived stress) did not differ between experimental conditions, *F*’s (1,89) < 2.03 *p’*s > 0.157. In addition, baseline cortisol did not differ between conditions *F*(1,87) = 0.27, *ns*. Analyses also indicated that negative affect and self-reported physiological arousal significantly increased from baseline to post-stressor for everyone, *F*(1,90) = 153.98, *p* < 0.001 and *F*(1,91) = 112.78, *p* < 0.001, respectively, indicating that the speech task was an effective stressor (see Supplementary Table S1 for descriptive statistics of all self-reported measures). In addition, the vast majority of participants described thoughts related to their interview after the 10-min rest period. Main analyses consisted of simple linear moderation modeling using Hayes’s PROCESS macro in SPSS (Hayes, 2013) to examine the effects of the experimental implicit mindfulness condition and the moderating role of trait mindfulness on post-stressor well-being while controlling for baseline levels of well-being. Both conditions were dummy coded with the control condition given a value of “0” and a value of “1” to the implicit mindfulness condition. Trait mindfulness was mean-centered to facilitate the interpretation of the simple and interaction effects (Aiken and West, 1991; Hayes, 2013). The product term between mean centered mindfulness scores and the condition term was computed to test for the trait mindfulness by condition interaction. The condition, trait mindfulness, and condition by trait mindfulness interaction terms, in addition to respective baseline levels of situational well-being used as covariates, were entered in the analyses described below.

### Situational Well-being

There was a significant simple effect of trait mindfulness on outcome levels of self-esteem, β = 0.51, *t*(87) = 4.16, *p* < 0.001, *r*^2^= 0.11, negative affect, β = -0.37, *t*(87) = -2.75, *p* = 0.007, *r*^2^= 0.06, self-reported physiological arousal, β = -0.32, *t*(87) = -2.24, *p* = 0.028, *r*^2^= 0.04, and perceived stress, β = -0.32, *t*(87) = -2.99, *p* = 0.004, *r*^2^= 0.04 (see **Table 1** for summary of results), indicating that for *participants in the control condition* (value of 0 for condition term), lower trait mindfulness was associated with lower levels of self-esteem, higher levels of negative affect, self-reported physiological arousal, and perceived stress following the speech task. There was a significant simple effect of condition on outcome levels of self-esteem, indicating that, at mean level of trait mindfulness (value of 0 on trait mindfulness term), participants in the implicit mindfulness condition reported significantly higher self-esteem than those in the control condition. Condition did not significantly predict other outcome measures of well-being, |β’s| < 0.15 |*t*’s| (87) < 1.59, *p*’s > 0.115.

**Table 1:** Regression coefficients for the simple and interaction effects from the simple linear moderation regression analyses.

Independent variables	Self-esteem		Negative affect		Self-reported physiological arousal		Perceived stress	
	β	*r*^2^	β	*r*^2^	β	*r*^2^	β	*r*^2^
Condition	0.16^∗^	0.03	-0.11		-0.14		-0.08	
MF	0.51^∗∗∗^	0.11	-0.37^∗∗^	0.06	-0.32^∗^	0.04	-0.32^∗∗^	0.04
Condition × MF	-0.29^∗^	0.04	0.35^∗∗^	0.05	0.31^∗^	0.04	0.20^∗^	0.02
Total *R*^2^ (*R*^2^ adjusted)	0.47 (0.45)		0.34 (0.31)		0.23 (0.20)		0.64 (0.63)	

*MF, trait mindfulness. β’s represent standardized regression coefficient. r^2^’s represent the square semi-partial correlation as a measure of effect sizes and are reported for significant effects only. Baseline measures of respective outcome measures were included as control variables in each regression analyses and are not reported here. ^∗^p < 0.05; ^∗∗^p < 0.01; ^∗∗∗^p < 0.001.*

**FIGURE 1:**
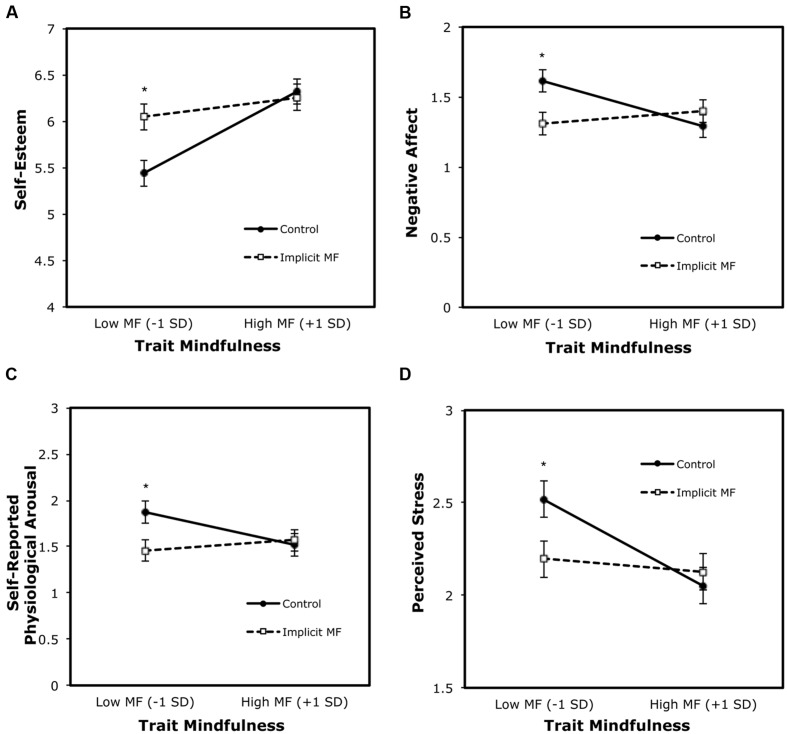
**Simple slopes of post-stressor situational well-being at 1 SD below and above mean trait mindfulness (MF) for Control and Implicit Mindfulness (Implicit MF) conditions. (A)** self-esteem; **(B)** negative affect; **(C)** self-reported physiological arousal; **(D)** perceived stress. Error bars represent standard error of the mean. ^∗^ represent condition difference at moderator level.

More importantly, and of most interest in our analyses, the condition by trait mindfulness interaction term significantly predicted outcome levels of self-esteem, β = -0.29, *t*(87) = -2.47, *p* = 0.015, *r*^2^= 0.04, negative affect, β = 0.35, *t*(87) = 2.62, *p* = 0.010, *r*^2^= 0.05, self-reported physiological arousal, β = 0.31, *t*(87) = 2.16, *p* = 0.033, *r*^2^= 0.04, and perceived stress, β = 0.20, *t*(87) = 2.09, *p* = 0.040, *r*^2^= 0.02, while controlling for their respective baseline levels. *Positive* affect was not significantly predicted by the condition × trait mindfulness interaction term, β = -0.11, *t*(87) = -0.88, *ns*. Tests of simple slopes (Aiken and West, 1991; Hayes, 2013) showed that participants with low trait mindfulness (at -1 standard deviation below mean trait mindfulness) in the implicit mindfulness condition reported significantly higher levels of situational self-esteem, β = 0.36, *t*(87) = 3.18, *p* = 0.002, *r*^2^= 0.06, lower levels of negative affect, β = -0.34, *t*(87) = -2.73, *p* = 0.008, *r*^2^= 0.06, less self-reported physiological arousal, β = -0.35, *t*(87) = -2.60, *p* = 0.011, *r*^2^= 0.06, and lower levels of perceived stress, β = -0.22, *t*(87) = -2.35, *p* = 0.021, *r*^2^= 0.02 than their counterparts in the control condition (see **Figure 1**). The simple slope analyses for participants with high trait mindfulness (at +1 standard deviation above mean trait mindfulness) were not significant (|β’s| < 0.12 |*t*’s| (87) < 0.99, *p*’s > 0.326).

### Cortisol

Cortisol was analyzed using a time-resolved fluorescence immunoassay (Dressendörfer et al., 1992) with reliability and validity support. In the current analysis, intra- and interassay coefficients variations were less than 10 and 12%, respectively, values under the recommended maximums of 12–15% (Nicolson, 2008). All cortisol measures were positively skewed and were therefore log transformed for our analyses. Preliminary analyses indicated that trait mindfulness did not act as a moderator in our cortisol analyses and was therefore dropped from future analyses.

**FIGURE 2:**
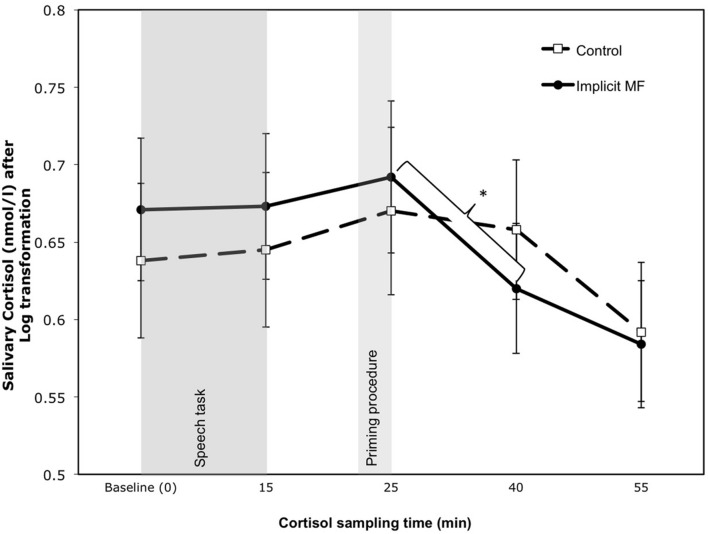
**Log transformed cortisol (nmol/l) at each sampling time for Control and Implicit Mindfulness (Implicit MF) conditions.** ^∗^ indicates significant decrease between T3 and T4 in Implicit MF condition. Error bars represent standard error of the mean.

To investigate the mitigating effects of priming mindfulness on cortisol recovery, our main analyses focused on the early stage cortisol recovery after peak cortisol reactivity and the semantic activation of mindfulness versus neutral words. We therefore conducted a mixed measures ANCOVA on cortisol measures taken during the 15-min period following peak reactivity and activation procedure (T3–T4, see **Figure 2**). The time (T3 vs. T4) by condition (mindfulness vs. control) mixed model ANCOVA with time as a within-subject factor while controlling for medication use, average number of cigarettes smoked in a day, time of last coffee, and baseline cortisol levels (T1) was conducted on log transformed cortisol measures. Results revealed only a significant time by condition effect, *F*(1,86) = 4.11, *p* = 0.046, η^2^= 0.05. Simple effects test revealed that for participants in the implicit mindfulness condition, there was a significant decrease in cortisol from T3 (*M*_T3_ = 0.68, *SE*_T3_ = 0.03) to T4 (*M*_T4_ = 0.62, *SE*_T4_ = 0.03), *F*(1,86) = 15.81, *p* < 0.001, η^2^ = 0.16, whereas there was no significant decrease for those in the control condition (*M*_T3_ = 0.69, *SE*_T3_ = 0.04; *M*_T4_ = 0.67, *SE*_T4_ = 0.03). The later stage recovery analysis of time (T4 vs. T5) by condition mixed model ANCOVA revealed a marginal main effect of time, *F*(1,86) = 3.34, *p* = 0.071, *η^2^* = 0.04, indicating that across both conditions, there was a marginal decrease from T4 to T5, but a non-significant time by condition effect, *F*(1,86) = 1.23, *ns.* The overall analysis of time (T1 to T5) by condition mixed model ANCOVA revealed non-significant main, *F*(4,348) = 1.9, *ns* and interaction effects, *F*(4,348) = 0.99, *ns.*

## Discussion

The current results show that implicitly activating mindfulness has beneficial psychological and physiological effects after undergoing a stressful speech task. Specifically, participants in the implicit mindfulness condition showed higher levels of situational self-esteem after the speech task compared to those in the control condition. In other words, whereas the social evaluative threat of the speech task significantly thwarted participants’ sense of self-worth in the control condition, those non-consciously primed with mindfulness were able to preserve their sense of self-esteem. In addition, the psychological benefits of non-consciously activating mindfulness seems to be most beneficial to those low in trait mindfulness, i.e., who do not habitually or routinely ‘pay attention, in a non-judgmental manner, to the present moment.’ Participants with low trait mindfulness showed higher levels of self-esteem, lower levels of negative affect and perceived stress, and lower levels of self-reported physiological arousal than their counterparts in the control condition. Together, these results indicate that unobtrusively activating mindfulness in participants with low trait mindfulness helps them recover from a social stress by momentarily engaging similar emotional-coping mechanisms as those with high dispositional mindfulness.

These results support other research conducted in our laboratory showing the stress-buffering effects of implicitly activating mindfulness and its potential mechanisms. Indeed, those in the implicit mindfulness condition reported greater positive emotions after failing at anagrams and recalling a very negative personal event, indicating an up-regulation of positive emotions (Bergeron and Dandeneau, in press). In the current study, implicitly activating mindfulness after experiencing a moderate social stressor attenuated participants’ negative affect, perception of stress, and self-reported physiological arousal, suggesting a down-regulation or buffering of negative arousal. The current results are also in line with many other studies showing that mindfulness and meditative training is associated with reduced perceptions of stress (Arch and Craske, 2010; Baer et al., 2012; Hoge et al., 2013; Creswell et al., 2014). They are also in line with Lutz et al.’s (2014) fMRI study showing that explicitly instructing participants to apply mindful awareness during specified trial was linked to an increase in activation of brain regions associated with emotion regulation and decreased activity in brain regions involved in the interpretation of negative stimuli. Lutz et al. (2014) interpreted their results as suggesting that the explicit activation of mindful awareness resulted in less arousal/autonomic activation while viewing negative stimuli. Our current results follow this logic and suggest that implicitly activating mindfulness attenuated one’s evaluation and recovery after a stressful experience.

The effect of implicitly activating mindfulness was also evident on participants’ physiological well-being. Results indicate that in the recovery stage following peak stress reactivity, those for whom mindfulness was non-consciously activated showed a greater decline in cortisol levels. This could indicate that the activation of mindfulness-related affective regulation, even at the non-conscious level, is a powerful factor in terminating HPA axis activation after stress, and returning the stress system to its baseline faster. These results are in line with Brown et al.’s (2012) study showing that higher trait mindfulness predicted lower cortisol reactivity on the TSST, however, whereas their results show the benefits of developing high dispositional mindfulness, the current results suggest that momentarily activating this innate capacity shows similar benefits. At first glance our results seem somewhat at odds with those of Creswell et al.’s (2014) study showing that a brief mindfulness meditation training decreased perceptions of psychological stress but *increased* cortisol reactivity to the TSST, and this especially for those with low trait mindfulness. Important methodological differences prevent us from directly comparing both studies, namely that the meditative training was administered before the TSST instead of during the recovery stage, however, visual inspection of their cortisol data indicate a similar accentuated decrease during the early stage recovery (equivalent to our analyses) after peak reactivity for those in the meditative training compared to those in the control training. Together, our results contribute to the growing literature showing the modulating effects of activating mindfulness (deliberately or automatically), on psychological and neuroendocrinological functioning.

Some authors have suggested that psychological flexibility is one of the mechanisms that contribute to the positive impacts of mindfulness practice and a mindful state of mind (Baer, 2010; Dunn et al., 2013). For example, Frewen et al. (2008) proposed that one’s capacity to let go of negative automatic thoughts may promote individuals’ cognitive flexibility thereby increasing ones’ potential range of responses to a given situation. Frewen’s results also suggest that the quality of automatic negative thoughts of individuals with greater levels of dispositional mindfulness report a greater capacity of letting go of their negative thoughts. This explanation may account for the moderating effect of trait mindfulness on all self-reported well-being measures in our study. The hypothetical job interview very likely triggered automatic negative thoughts for everyone – as supported by our manipulation check data – and supporting Frewen et al.’s (2008) results. Participants with high trait mindfulness in neither condition differed on measures of psychological well-being presumably because in both cases, their potential range of responses and cognitive flexibility allowed them to reappraise the negative impacts of the speech task in a more constructive, non-judgmental, and positive light. Most interesting, however, is that individuals with low trait mindfulness for whom mindfulness was implicitly triggered, showed similar responses. We believe that by unobtrusively activating mindfulness, participants with low trait mindfulness reacted to a stressful experience by likely disengaging from their habitual emotional thought patterns and processed their experience in a psychologically more flexible manner thereby allowing them to see themselves with greater value (self-esteem), experiencing less negative affect, and less perceived stress than usual. Testing the mediating role of psychological flexibility in future studies would be of great interest.

Certain methodological limitations of the present study should be considered. First, the implicit nature of the mindfulness activation procedure makes it very difficult to measure whether mindfulness was truly activated. The mere exposure to self-reported state mindfulness questions (as a post manipulation check) would provide explicit exposure to the concept and thereby confound the effects of the implicit priming. Also, mindfulness is a bourgeoning topic and researchers are starting to better understand its underlying mechanisms. However, it still remains unclear exactly which component or components of mindfulness act on which mechanism – and more importantly for whom these mechanisms are of most benefit. It would be important for future studies to examine the mechanistic functioning of this novel pathway to mindfulness and whether the active mechanisms differ from those involved in deliberate meditative training.

## Conclusion

Our results suggest that non-consciously activating people’s innate capacity for mindfulness can help people better recover from a moderate stressor, especially when one is not in the habit of ‘paying attention in a non-judgmental manner to the present moment.’ In support of the growing evidence for the stress-buffering benefits of trait mindfulness, our results also suggest that future research looking at the benefits of non-consciously activating one’s innate capacity for mindfulness may provide a novel perspective on the underlying processes of mindfulness.

## Author Contributions

CB contributed to the design of the study, acquisition, analysis and interpretation of the data, and drafting and revising of the intellectual content. IA-D contributed to design of the study, the acquisition and interpretation of the data, and drafting of the work. SD contributed to the conception and design of the study, analysis and interpretation of the data, revising of the content and final approval of the version to be published and is accountable for all aspects of the work. We thank Jens Pruessner for help with cortisol analyses and sampling in addition to valuable comments on drafts of the manuscript.

## Conflict of Interest Statement

The authors declare that the research was conducted in the absence of any commercial or financial relationships that could be construed as a potential conflict of interest.
