# Supplementary material for: “Letting Go” (Implicitly): Priming Mindfulness Mitigates the Effects of a Moderate Social Stressor
Source: Front Psychol. 2016 Jun 13;7:872. doi: 10.3389/fpsyg.2016.00872 (PMC4904283; doi:10.3389/fpsyg.2016.00872)
Supplement: Supplementary file 1 [file Table_1.PDF]

SUPPLEMENTAL MATERIAL

“Letting Go” (Implicitly): Priming Mindfulness Mitigates the Effects of a Moderate Social Stressor

Catherine M. Bergeron, Isabelle Almgren-Doré, and Stéphane Dandeneau
Department of Psychology, Université du Québec à Montréal, Québec, Canada

Public Speaking Task

For the speech task, participants were told they would have 10 minutes to prepare a presentation explaining why they would be the best candidate for a hypothetical job. They were also informed that their 5-minute speech would be delivered in front of a ‘hiring manager’, who would ask follow-up questions for clarifications. Before leading the participant into the interview room, the research assistant mentioned that notes could be taken during the preparation period and informed participants that the video camera and a microphone in the room would only be turned on during the interview. Finally, to increase self-awareness, participants were asked to practice in front of a full-length mirror. Following the 10-minute preparation period, the hiring manager (a 30 year old female dressed in business like attire) entered the interview room, turned on the video camera and microphone, and took away the participant’s notes. Participants were then instructed to stand in front of the microphone and begin their 5-min speech. This procedure was chosen because its social-evaluative component has a reliable effect on the hypothalamic-pituitary-adrenal axis (HPA axis), responsible for cortisol secretion (Dickerson & Kemeny, 2004).

Validation of Mindfulness prime words supplemental material

The words chosen to represent the concept of “mindfulness” underwent an initial validation phase in order ensure that the focus of the priming procedure was on mindfulness and *not* on general positive concepts (e.g. acceptance is a concept related to mindfulness that also has positive connotations). A total of 41 independent evaluators rated the positive and negative valence of the set of 8 mindfulness words, the 8 neutral used in the control condition and final set of 8 positive words (e.g. smile, happiness, health) not used in either conditions but used here as a “positive” reference point. All words were rated on the following two 100-point slider scales: “Not at all *negative* (0)” to “Very *negative* (100)” and “Not at all *positive* (0)” to “Very *positive* (100)”. The presentation of the negative and positive evaluations was counterbalanced across evaluators, i.e. half rated the negative valence then the positive valence and the other half positive followed by negative valences. Next, evaluators were presented with the set of 8 mindfulness words and asked to describe, in an open-ended question, what thoughts, concepts, and ideas these words elicited. Finally, evaluators were asked to rate their description of the set of mindfulness words along 7 mindfulness-related dimensions (e.g. resistance vs. acceptance, elsewhere vs. present, indifferent vs. sensitive, inattentiveness vs. attentiveness, forgetful vs. mindful, denying vs. observing,

critical vs. acceptant) by indicating on a 100-point slider scale to which anchor, e.g., resistance (0) or acceptance (100), their description most belonged.

Along the *positive* valence evaluation, analyses indicate that there was no difference between mindfulness words (M = 63.41, SD = 28.35) and neutral words (M = 56.64, SD = 25.85), $t(40)$ = -1.67, *ns.,* but that mindfulness words were rated as significantly *less* positive than positive words (M = 81.47, SD = 27.23), $t(40)$ = -5.50, $p < .001$, r = .66. Along the *negative* valence evaluation, mindfulness words (M = 17.02, SD = 18.55) were rated as significantly *more* negative than positive words (M = 6.82, SD = 15.50), $t(40)$ = 5.66, $p < .001$, r = .67, and more negative than neutral words (M = 12.72, SD = 17.85), $t(40)$ = 2.21, $p < .05$, r = .33. We also tested the positivity ratio (positive – negative) of each category of words whereby a high ratio indicates a greater positive rating relative to negative rating. Results indicate that the positivity ratio for mindfulness words (M = 46.38, SD = 39.63) was significantly lower than that for positive words (M = 74.65, SD = 37.25), $t(40)$=-7.19, $p < .001$, r = .75 and did not significantly differ from the positivity ratio of neutral words (M = 43.92, SD = 34.66), $t(40)$ = .571, *ns.* One-sample *t*-tests were conducted on each mindfulness-related dimensions, with 50 as test point, to investigate the interpretation of the list of mindfulness words. The list of mindfulness words were rated, on all 7 dimensions, as significantly greater than the 50 test point, *t's* = 3.21 to .8.28 with *p*'s < .01, indicating that our mindfulness primes were more related to mindfulness (e.g. acceptance, sensitive, mindful) than non-mindfulness (e.g. resistance, forgetful, critical).

Interestingly, in the open-ended question asking evaluators to describe their thoughts, concepts and ideas, no evaluator explicitly mentioned the word 'mindfulness' per se indicating that the concept of *mindfulness* may not be explicitly or deliberately associated with these words but rather that the fundamental ideas and concepts related to mindfulness are nonetheless activated. Many evaluators used terms like 'accepting oneself' and 'being in the present moment' to refer to the general idea of mindfulness. In sum, the results from the validation procedure indicate that the words used to nonconsciously activate mindfulness a) are not priming a positive concept per se and b) are related to mindfulness-related dimensions.

Supplemental Table 1

Baseline and Post-Stressor Means (Standard Deviations) of Self-Reported Dependent Measures

	Control condition		Implicit Mindfulness condition	
Dependent measures	Baseline	Post	Baseline	Post
Self-esteem	5.78 (.96)	5.81 (.99)	6.02 (.59)	6.20 (.68)
Negative affect	1.49 (.42)	1.47 (.50)	1.43 (.38)	1.34 (.40)
Self-reported physiological arousal	1.91 (.73)	1.72 (.65)	1.72 (.56)	1.50 (.54)
Perceived stress	2.27 (.75)	2.29 (.79)	2.23 (.75)	2.16 (.71)
